# Serum and urine concentrations of morphine and morphine metabolites in patients with advanced cancer receiving continuous intravenous morphine: an observational study

**DOI:** 10.1186/s12904-015-0052-9

**Published:** 2015-10-27

**Authors:** Yong Joo Lee, Sang-Yeon Suh, Junghan Song, Sanghee Shiny Lee, Ah-Ram Seo, Hong-Yup Ahn, Myung Ah Lee, Chul-Min Kim, Pål Klepstad

**Affiliations:** Department of Palliative Medicine, Seoul St. Mary’s Hospital, College of Medicine, The Catholic University of Korea, Seoul, South Korea; Department of Medicine, Dongguk University School of Medicine, 30 Pildong-ro 1-gil, Jung-gu, Seoul, 100-715 South Korea; Department of Laboratory Medicine, Seoul National University Bundang Hospital, Seoul National University College of Medicine, Seoul, South Korea; Department of Statistics, Dongguk University, Seoul, South Korea; Division of Oncology, Department of Internal Medicine, Seoul St. Mary’s Hospital, College of Medicine, The Catholic University of Korea, Seoul, South Korea; Department of Family Medicine, Seoul St. Mary’s Hospital, College of Medicine, The Catholic University of Korea, Seoul, South Korea; Departments of Anaesthesiology and Intensive Care Medicine, St. Olvas University Hospital, Trondheim, Norway; Department of Circulation and Medical Imaging, Medical Faculty, Norwegian University of Technology and Science, Trondheim, Norway

**Keywords:** Morphine, Metabolite, Serum, Urine, Monitoring, Advanced cancer

## Abstract

**Background:**

The feasibility and clinical implication of drug monitoring of morphine, morphine-6-glucuronide (M6G) and morphine-3-glucuronide (M3G) need further investigation. This study aimed to determine what predicts serum concentrations of morphine in cancer patients receiving continuously intravenous morphine, the relationships between serum concentration of morphine/its metabolites and urinary concentrations, and the relation between morphine concentrations and with clinical outcomes.

**Methods:**

We collected serum and urine samples from 24 patients with advanced cancer undergoing continuously intravenous morphine therapy. Serum samples were obtained at day one. Spot urine samples were collected once daily on three consecutive days. Pain and adverse drug events were assessed using the Korean version of MD Anderson Symptom Inventory.

**Results:**

A total of 96 samples (72 urine and 24 serum samples) were collected. Median dose of morphine was 82.0 mg/24 h. In a multivariate analysis, total daily morphine dose was the most significant predictors of both serum and urine concentration of morphine. Morphine, M6G, and M3G in serum and urine were statistical significantly correlated (correlation coefficient = 0.81, 0.44, 0.56; *p* values < 0.01, 0.03, 0.01, respectively).

**Conclusion:**

Spot urine concentrations of morphine and its metabolites were highly correlated to those of serum. Total dose of daily morphine was related to both serum and urine concentration of morphine and its metabolites.

## Background

Morphine is the most used treatment for moderate or severe pain in advanced cancer patients. However, there has been stigma related to morphine among patients and their families. Many patients receiving morphine encounter adverse events of morphine such as drowsiness and nausea, and in extreme cases morphine leads to opioid-induced neurotoxicity (OIN) [1]. One distinctive feature of morphine responsiveness is that there are high inter-individual variations in the needed dose among patients [2]. Previous studies suggest that variable bioavailability, metabolism and elimination of morphine and its metabolites might explain some of the variability in doses [3]. Morphine is metabolized into morphine-3-glucuronide (M3G) and morphine-6-glucuronide (M6G). M6G has been suggested to have higher analgesic potency than morphine, and may cause nausea and sedation [4]. M3G has been reported to exert an antianalgesic effect, and is suggested to elicit neurotoxicity [5]. However, the relation between morphine metabolites and clinical symptoms is still controversial [6–8]. In previous studies, most patients use oral morphine, and most studies are performed in one ethnic group, Caucasians. Therefore, a study including non-Caucasian patients would be of interest. Thus, this study aimed to investigate in Korean inpatients with advanced cancer receiving continuously intravenous morphine; 1) What predicts serum and urine concentration of morphine and its metabolites, and 2) What is the relationship between serum and urinary concentrations of morphine.

## Methods

### Study design

This was a prospective observational study for inpatients with advanced cancer of a university hospital in South Korea. The institutional review board (IRB) of the study hospital approved this study (IRB Number: KC12TIS10076). All patients participated in this study provided written informed consents.

### Study participants

Twenty-four Korean patients with far advanced cancer who were admitted to Seoul St. Mary’s hospital from July 2012 till October 2013 were enrolled in this study. Patients who were more than 18 years old receiving continuous intravenous morphine treatment for more than three days were included. To minimize variability due to gastrointestinal absorption and first-pass metabolism, we only included patients receiving intravenous morphine. We excluded patients who were unable to answer to questions regarding pain, adverse events and health status. Patients who had alterations in consciousness caused by organic brain diseases such as dementia, stroke or, brain metastasis were excluded. Patients with anuria (urine output <100 ml/day), bowel obstruction (based on clinical and radiologic findings), liver dysfunction (alanine transaminase ≥100 IU/l) and renal dysfunction (serum creatinine ≥ 2.0 mg/dl) were also excluded.

### Assessments of clinical variables

At inclusion age, gender, and cancer type were registered. Patients’ functional status was assessed using Karfnosky performance status. Alanine aminotransferase and creatinine measurements were determined using standard analytical methods. The daily amount of intravenous fluid, daily urine output, daily total dosage of morphine were registered for all three days of the study.

Pain, adverse events of morphine (drowsiness, nausea, confusion) and health status were rated by the patients using numeric rating scale (0 ~ 10). Items for drowsiness and nausea were adopted from the symptom scales of MD Anderson symptom inventory-Korean (MDASI-K). The scales describe patient’s symptoms during the last 24 h, with 0 being “not at all” while 10 being “as bad as you can imagine”. A research nurse explained confusion as ‘decrement of concentration ability, difficulty in paying attention’ during the interview. Health status was checked using a self-rated item from 0 representing “worst imaginable health status” to 100 representing “best imaginable health status”.

### Samples and analyses of blood and urine

We obtained urinary samples besides blood samples in order to observe if urine concentrations of morphine and metabolites reflect serum concentrations. Blood samples to measure serum concentrations of morphine and its metabolites were drawn during the routine morning collection of blood tests. EDTA tubes were used, and serum was separated by centrifugation and stored at −70 °C. We obtained serum samples at day 1 and three consecutive urine samples from day one to day three (D3) of the study. Urine samples were gathered as the first spot urine in the morning. Urine samples were put in tubes with no additives and stored at −70 °C. All samples were analyzed at the Department of Laboratory Medicine, Bundang Seoul National University (Gyeonggido, South Korea). Samples were analyzed for concentrations of morphine, M6G and M3G by using ultra performance liquid chromatography-tandem mass spectrometry (Waters Aquity UPLC Xevo TQ-S, Waters, Watford, United Kingdom). Its limits of detection were: 2.5 ng/ml for morphine, 2.0 ng/ml for M6G, and 2.0 ng/ml for M3G. The analytical coefficients of variation were: 5.0–5.2 % for morphine, 6.4–6.9 % for M6G, and 5.9–7.0 % for M3G.

### Statistical analysis

Results were shown in medians and ranges. Correlations of urine and serum concentrations was examined using Spearman method. The relationships of morphine dose and its serum and urine concentrations were evaluated by linear regression analysis. Interpatient variations in morphine concentrations were investigated by calculating the individual urine/serum ratios and creating box plots for the ratios. The relationship between serum and urine concentrations and clinical outcomes were analyzed with the Spearman correlation. Analyses were conducted using IBM SPSS version 21 (Statistical Package for Social Services, Chicago, IL). Statistical significance was defined as a *p*-value < 0.05.

## Results

### Baseline characteristics of patients

The median age of patients was 62.5 years and 75 % (18/24) were males (Table 1). The most frequent site of cancer was colon/rectum, pancreas and stomach in a descending order. Patients’ median Karnofsky performance status was 60. The reason for admission was mainly for pain control and supportive care with the exception of three patients who were admitted for chemotherapy. All patients received morphine for at least three days prior to study enrollment. The median of total dose of intravenous morphine during the study period was 82 mg/day (D1, D3) and 153.4 mg/day (D2). The median of administered intravenous fluid amount was 1050 ~ 1210 ml/day while that of urine output was 1500 ml/day. The patients self-reports of symptoms are given in Table 1.

**Table 1:** Demographic and clinical characteristics of study participants (*n* = 24)

	n ( %)
Gender	
Male	18 (75)
Female	6 (25)
Age (years)	62.5 (38, 80)^a^
Karnofsky Performance Status	60 (50, 80)^a^
Cancer type	
Stomach	4 (16.7)
Colon /Rectum	11 (45.8)
Ovary/Cervix	1 (4.2)
Liver	2 (8.3)
Pancreas	5 (20.8)
Kidney/Bladder	1 (4.2)
Thyroid	1 (4.2)
Total intravenous morphine dose (mg/day)	
Day 1	82 (14, 1000)^a^
Day 2	153.5 (2, 1120)^a^
Day 3	82.0 (0, 1132)^a^
Pain (NRS, 0 ~ 10)	
Day 1	5.0 (0, 9)^a^
Day 2	5.0 (0, 7)^a^
Day 3	4.5 (0, 9)^a^
Drowsiness (NRS, 0 ~ 10)	
Day 1	5.0 (0, 10)^a^
Day 2	5.0 (0, 10)^a^
Day 3	3.5 (0, 10)^a^
Confusion (NRS, 0 ~ 10)	
Day 1	5.0 (0, 10)^a^
Day 2	3.0 (0, 10)^a^
Day 3	3.0 (0, 10)^a^
Nausea (NRS, 0 ~ 10)	
Day 1	2.0 (0, 10)^a^
Day 2	0.0 (0, 10)^a^
Day 3	1.0 (0, 10)^a^
Health status (NRS, 0 ~ 10)	
Day 1	5.0 (0, 10)^a^
Day 2	6.0 (0, 9)^a^
Day 3	5.5 (0, 9)^a^
Total intravenous fluid (ml/day)	
Day 1	1106.5 (200, 2019)^a^
Day 2	1210.5 (0, 2088)^a^
Day 3	1050.0 (0, 2240)^a^
Total urine output (ml/day)	
Day 1	1500.0 (490, 2900)^a^
Day 2	1500.0 (600, 3300)^a^
Day 3	1550.0 (250, 3450)^a^
Laboratory variables	
Alanine transaminase (IU/L)	23 (6, 75)^a^
Creatinine (mg/dl)	0.7 (0.4, 1.7)^a^

^a^ Value represents median (minimum, maximum)

*NRS* numeric rating scale

### Serum and urine concentrations of morphine, M6G, M3G and metabolic ratios

On D1, the median of morphine concentration was 268.8 nmol/liter in the serum and 33293.9 nmol/liter in the urine (Table 2). On D1, the M6G concentration was 216.7 nmol/liter in the serum and 11485.3 nmol/liter in the urine. The M3G concentration on D1 was 1036.6 nmol/liter in the serum and 34780.8 nmol/liter in the urine. The ratio of M6G/M was 0.62 in the serum and 0.82, 0.76, 0.64 in the urine on D1, D2, and D3, respectively. The ratio of M3G/M was 3.12 in the serum and in urine 1.70, 1.65, 1.97 on D1, D2, D3, respectively (Table 2).

**Table 2:** Serum and urine concentrations of morphine, metabolites, and metabolic ratios (*n* = 24)

	Median	Mean	Range
Serum			
Day1			
M (nmol/l)	268.8	5617.8	35.0–70302.6
M6G (nmol/l)	216.7	613.6	21.7–5229.1
M3G (nmol/l)	1036.6	2372.8	108.4–24160.2
M6G/M (nmol/l)	0.6	1.6	0.0–9.3
M3G/M (nmol/l)	3.1	5.0	0.1–21.5
Urine			
Day1			
M (nmol/l)	33293.9	253924.0	1051.4–1380117.6
M6G (nmol/l)	11485.3	31195.6	1267.3–268278.9
M3G (nmol/l)	34780.8	81053.0	3828.9–518352.5
M6G/M (nmol/l)	0.8	0.8	0.0–2.0
M3G/M (nmol/l)	1.7	1.9	0.1–5.6
Day2			
M (nmol/l)	23130.5	407484.6	1401.8–3078804.8
M6G (nmol/l)	10726.8	72322.2	1733.6–1177133.4
M3G (nmol/l)	37272.8	227898.7	4664.5–4007047.2
M6G/M (nmol/l)	0.8	0.7	0.0–1.3
M3G/M (nmol/l)	1.7	1.7	0.1–4.0
Day3			
M (nmol/l)	19625.9	122126.5	350.5–2695750.3
M6G (nmol/l)	12568.8	56816.3	650.1–686950.1
M3G (nmol/l)	32938.8	460521.7	2600.4–1372594.1
M6G/M (nmol/l)	0.6	0.9	0.0–4.7
M3G/M (nmol/l)	2.0	2.6	0.1–15.2

*M* Morphine, *M6G* Morphine-6-glucuronide, *M3G* Morphine-3-glucuronide

### Correlation of serum and urine concentrations

All concentrations of morphine, M6G and M3G in the serum were significantly correlated to its urine concentration (Table 3).

**Table 3:** The correlation coefficients of morphine/morphine metabolites/metabolic ratios between serum and urine (*n* = 24)

	M (nmol/liter)	M6G (nmol/liter)	M3G (nmol/liter)	M6G/M	M3G/M
Coefficient (*p*-value)	0.81(<0.01)	0.44 (0.03)	0.56 (0.01)	0.82 (<0.01)	0.76 (<0.01)

*M* Morphine, *M6G* Morphine-6-glucuronide, *M3G* Morphine-3-glucuronide

*P*-values are derived from spearman rank correlation

### Clinical characteristics and concentrations of morphine and metabolites

Daily morphine dose was significantly correlated to concentrations of morphine and metabolites concentrations in both serum and urine (Table 4) and multiple linear regression showed that total daily morphine dose explained the majority of the variability of serum and urine concentrations as noted from the low R-square values when omitting morphine dose from the models (Table 5). We observed no significant correlation between serum or urine morphine or metabolite concentrations with clinical outcomes (data not shown)

**Table 4:** The correlation coefficients between total morphine dose and morphine concentration, and its metabolites in serum and urine (*n* = 24)

	M	M6G	M3G
Serum (Day1)	0.53 (0.01)	0.58 (0.00)	0.72 (<0.01)
Urine (Day1)	0.44 (0.03)	0.68 (<0.01)	0.65 (<0.01)
Urine (Day2)	0.47 (0.02)	0.46 (0.02)	0.51 (0.01)
Urine (Day3)	0.55 (0.01)	0.76 (<0.01)	0.65 (<0.01)

*M* Morphine, *M6G* Morphine-6-glucuronide, *M3G* Morphine-3-glucuronide

*P*-values are parenthesized and derived from spearman rank correlation

**Table 5:** Linear regression model of factors predictive of serum and urine concentrations of morphine (*n* = 24)

Outcome	Factors Contributing to outcome	Estimates for individual factors with model 1		Estimate for model 1	Estimate for model 2
		Coef.	*P*-value	Adjusted *R* ^2^	Adjusted *R* ^2^
Serum					
M (Day 1)	Morphine dose (mg/24 h)	0.60	0.01	0.33	0.08
Urine					
M (Day 1)	Morphine dose (mg/24 h)	0.52	0.61	0.10	0.14
	Age	−2.05	0.06		
	Gender	−2.02	0.06		
M (Day 2)	Morphine dose (mg/24 h)	0.97	0.00	0.40	−0.05
	Creatinine	−0.44	0.07		
	Gender	−0.48	0.03		
M (Day3)	Morphine dose (mg/24 h)	0.58	0.01	0.56	0.29

Estimated Model 1: Independent factors in all analyses were morphine dose, gender, daily intravenous fluid amounts, creatinine, ALT, and age

Estimated Model 2: Independent factors in all analyses were gender, daily intravenous fluid amounts, creatinine, ALT, and age (Morphine dose was omitted from Model 1)

As for Urine model, daily urine output was used instead of daily intravenous fluid amounts in each model

*Coef* standardized coefficient in regression model, *M* Morphine concentration (nmol/liter), *ALT* alanine transaminase

## Discussion

Our study revealed that spot urine concentrations of morphine and its metabolites were highly correlated to those in the serum, and that morphine dose was the important predictor of both serum and urine concentrations of morphine.

This study was different from previous studies in terms of subjects and the route of drug administration. Our subjects were all inpatients undergoing morphine therapy through the intravenous route. Intravenous route omits variable gastric absorption, and it is not influenced by first pass metabolism. In addition to this, all patients had received a continuous intravenous infusion of morphine for more than three days prior to study commencement ensuring stable serum morphine concentrations. Moreover, we measured both concentrations of morphine/metabolites in serum and urine samples.

To the best of our knowledge, this is the first study that investigated the use of urine samples with serum samples for in morphine drug monitoring in cancer patients. Our study findings highlight the usefulness of urine test in patients undergoing intravenous morphine therapy. The advantages of urine test are its technical simplicity, painless collection and inexpensiveness [9, 10]. The urine samples in our study were only morning spot urine and not collected throughout a 24-h period. In spite of the simple method, our urine test results showed significant relationships with the corresponding serum observations. Interestingly, one recent study compared opioid concentrations of oral fluid with those of serum [11]. This study reported that oral fluid was a valid substitute for serum sampling for patients on chronic oral morphine therapy. We believe that urine analysis is even more familiar to the patients.

We confirmed that the total dose of daily morphine was related to the both serum and urine concentration of morphine, M6G and M3G. A similar relationship was demonstrated by Klepstad et al. [12], while a recent study showed a poor correlation between morphine dose and serum concentrations [13]. There are at least two factors that may explain such an inconsistency. First, there is a well-known inter-individual variability in the pharmacokinetics of morphine. The wide inter-individual variability including outliers reduce correlation between dose and serum, concentrations. Second, is the different route of administration for morphine. In our study, the correlation was strong and significant despite of its small sample size probably because the intravenous route eliminated variability due to intestinal absorption or first pass effect.

According to a systematic review, the weighted mean ratios in serum between morphine and metabolites were 6 (range: 0.2–15) for M3G:M and 0.9 (range: 0.03–2.6) for M6G: M in patients with normal renal function given intravenous morphine [14] compared to ratios in serum of 3.1 for M3G: M and 0.6 for M6G: M in our study. However, the ratios in our study is within the range reported by Faura et al. suggesting that limited sample size in our and others studies may explain some variability in numbers between studies. We observed that the urine ratios were 1.7–2.0 and 0.6–0.8 for M3G:M and M6G:M, respectively. Urine ratios are not available in previous studies. Thus, we could not compare our data to others.

While monitoring of concentrations of morphine and morphine metabolites is generally not believed to be important for routine drug monitoring [6], it can be useful for detecting accumulation of morphine and morphine metabolites. High serum concentrations of opioids is known to be associated with fatal opioid overdoses [15, 16]. This is particularly relevant to patients with renal dysfunction who are at higher risk of developing opioid toxicities due to reduced clearance of active metabolites. Some investigators have suggested that increased M3G concentrations may cause neuroexitatory adverse effects [5], have an anti-analgesic effect [17], or cause nausea in patients with renal failure [3]. Furthermore, dehydration, drug interactions and infections may increase accumulation of metabolites, especially in elderly patients [1]. Therefore, drug monitoring using a non-invasive method during opioid therapy might be valuable in examining unexpected clinical effects. However, its use in clinical practice is currently unestablished and remains limited to research purposes.

We recognize that this study has some limitations. Firstly, because our patients were a small and heterogenous group of advanced cancer patients, this study may be unable reveal meaningful relationships between symptoms and drug/metabolites concentrations. Secondly, serum concentrations of morphine metabolites are known to be higher in patients with renal impairment and thus these patients have a higher risk of nausea, vomiting and confusion [3]. Because patients with renal failure were excluded from our study, relationships relevant for cancer patients with renal failure were not detected in our analyses. Thirdly, one may raise a question about the wide range of morphine dosages in our study subjects. At the time of study enrollment, our patients had been receiving intravenous morphine more than three days to ensure a steady state of morphine and prior to this, all patients had received either oral or intravenous morphine. Therefore, all patients had used morphine for some time and were as expected titrated to different doses of morphine reflecting the known large interindividual dose variability of morphine given for cancer pain. Finally, the numbers of patients were relatively low in this study. However, the total number of samples was ninety-six due to repeated measurements. One of the study’s strengths was that variability of pharmacokinetic observation was lower because of that all patients received morphine intravenously. Furthermore, this study is the first study on morphine pharmacokinetics during chronic morphine therapy for cancer pain in ethnic Koreans. Our findings would promote further similar studies using non-invasive methods such as urine tests, and we also suggest similar investigations for other opioids (e.g., fentanyl and oxycodone).

## Conclusion

Spot urine concentrations of morphine and its metabolites were highly correlated to those of serum. Urine tests can be considered if evaluations of morphine/metabolites levels are needed in patients receiving continuous intravenous morphine. Clinical use of routine monitoring still requires further investigations.

## Abbreviations

M: Morphine
M6G: Morphine-6-glucuronide
M3G: Morphine-6-glucuronide
